# Imputation of the continuous arterial line blood pressure waveform from non-invasive measurements using deep learning

**DOI:** 10.1038/s41598-021-94913-y

**Published:** 2021-08-03

**Authors:** Brian L. Hill, Nadav Rakocz, Ákos Rudas, Jeffrey N. Chiang, Sidong Wang, Ira Hofer, Maxime Cannesson, Eran Halperin

**Affiliations:** 1 Department of Computer Science, University of California, Los Angeles, CA USA; 2 Department of Computational Medicine, University of California, Los Angeles, CA USA; 3 Department of Statistics, University of California, Los Angeles, CA USA; 4 Department of Anesthesiology and Perioperative Medicine, David Geffen School of Medicine At UCLA, Los Angeles, CA USA; 5 Department of Human Genetics, University of California, Los Angeles, CA USA

**Keywords:** Medical research, Machine learning, Predictive medicine

## Abstract

In two-thirds of intensive care unit (ICU) patients and 90% of surgical patients, arterial blood pressure (ABP) is monitored non-invasively but intermittently using a blood pressure cuff. Since even a few minutes of hypotension increases the risk of mortality and morbidity, for the remaining (high-risk) patients ABP is measured continuously using invasive devices, and derived values are extracted from the recorded waveforms. However, since invasive monitoring is associated with major complications (infection, bleeding, thrombosis), the ideal ABP monitor should be both non-invasive and continuous. With large volumes of high-fidelity physiological waveforms, it may be possible today to impute a physiological waveform from other available signals. Currently, the state-of-the-art approaches for ABP imputation only aim at intermittent systolic and diastolic blood pressure imputation, and there is no method that imputes the continuous ABP waveform. Here, we developed a novel approach to impute the continuous ABP waveform non-invasively using two continuously-monitored waveforms that are currently part of the standard-of-care, the electrocardiogram (ECG) and photo-plethysmogram (PPG), by adapting a deep learning architecture designed for image segmentation. Using over 150,000 min of data collected at two separate health systems from 463 patients, we demonstrate that our model provides a highly accurate prediction of the continuous ABP waveform (root mean square error 5.823 (95% CI 5.806–5.840) mmHg), as well as the derived systolic (mean difference 2.398 ± 5.623 mmHg) and diastolic blood pressure (mean difference − 2.497 ± 3.785 mmHg) compared to arterial line measurements. Our approach can potentially be used to measure blood pressure continuously and non-invasively for all patients in the acute care setting, without the need for any additional instrumentation beyond the current standard-of-care.

## Introduction

Each year in the United States, 5.7 million patients are admitted to an intensive care unit (ICU) and nearly 50 million patients undergo surgery. Hypotension and hypertension in the ICU and perioperative period are associated with adverse patient outcomes including stroke^1^, myocardial infarction^2^, acute kidney injury^3^, and death^4^. Recent studies even suggest that only a few minutes of hypotension in the acute care setting increases the incidence of these complications^5^. These observations strongly suggest that continuous blood pressure monitoring is critical in the acute care setting to identify periods of hypertension and/or hypotension as early as possible. Today, the gold standard for blood pressure monitoring is the invasive arterial line, a small catheter inserted into an artery, which enables continuous blood pressure monitoring^4^. However, this technique is highly invasive and is associated with significant complications such as bleeding, hematoma, pseudoaneurysm, infection, nerve damage, and distal limb ischemia^6, 7^, and thus it is only applied to very high-risk patients.

On the other hand, the widely used non-invasive blood pressure monitoring system using cuff-based devices is both inaccurate and intermittent, only allowing for the monitoring of blood pressure every three or five minutes^8^. More recently, devices allowing for continuous and non-invasive blood pressure monitoring have been introduced. These devices, however, are sensitive to patient movement, they are expensive, and they cause continuous pressure on the finger that can interfere with blood circulation^9^. Additionally, the accuracy of the device can deteriorate in patients with severe vasoconstriction, peripheral vascular disease, or distorted fingers due to arthritis^10^.

The need for measuring the continuous blood pressure non-invasively suggests that the physiological waveform of the blood pressure should be imputed from other data. However, previous attempts at blood pressure imputation have primarily focused on the imputation of discrete systolic and diastolic blood pressure measurements taken intermittently by cuff^11–14^ or at the resolution of a heartbeat^15–17^, while high-risk patients need to be monitored using the continuous arterial line blood pressure *waveform,* and thus current methods are not adequate for usage in critical care settings. Moreover, many of the previously-studied cohorts consisted of healthy patients at rest, and therefore the blood pressure variability is not as dynamic compared to patients in the ICU. This calls into question the utility of these approaches in real life settings. Finally, many of the existing methods developed and tested their models in the same set of patients by training the model on an earlier part of each patient record and testing the model on the remaining data. Additionally, the number of patients used was often only several dozen, all coming from the same health system. This calls into question the generalizability of the models to unseen patients. For example, Sideris et al. impute the arterial blood pressure waveform by training the model on the same patient for which arterial blood pressure waveforms are provided^18^. However, such a scenario is not clinically useful, since applying such an approach will require invasive monitoring of the patients for at least part of the time.

In this paper, we present the development, training, and validation of a novel non-invasive and continuous deep learning method for predicting the arterial blood pressure waveform using the ECG waveform, the pulse oximeter (PPG) waveform, and non-invasive blood pressure cuff measurements. These measurements are collected as part of the current standard-of-care, and therefore no additional patient monitoring devices are needed. Our method leverages a well-known deep learning model architecture originally designed for image segmentation (V-Net^19^), and we adapted it for 1D physiological waveform signals. A key aspect of our preprocessing pipeline includes a manual labeling of PPG quality in a large subset of the training data to improve the signal-to-noise ratio and remove artifacts. The manual labeling was used to train a deep neural network to predict the signal quality of the waveform, resulting in a high-quality preprocessing pipeline that can be used beyond the scope of this study. We demonstrate that the modified 1D V-Net approach provides a highly accurate prediction of continuous arterial blood pressure waveform (root mean square error 5.823 (95% CI 5.806–5.840) mmHg), as well as the derived systolic (mean difference 2.398 ± 5.623 mmHg) and diastolic blood pressure (mean difference − 2.497 ± 3.785 mmHg).

As opposed to previous studies, here we show that the modified 1D V-Net approach successfully generalizes to new patients. Particularly, we validate the approach on non-healthy populations from ICUs in two different health systems, and our training and validation cohorts include different sets of patients.

## Results

### Description of dataset and features

Two separate cohorts of ICU patients were used in this study to train and validate the method. The first cohort consisted of randomly sampled ICU patients from the Medical Information Mart for Intensive Care version III (MIMIC-III)^20^ waveform database who had ECG waveforms, photo-plethysmographic (PPG) waveforms, arterial blood pressure (ABP) waveforms, and at least one non-invasive blood pressure measurement. After exclusion of patients with insufficient data, the remaining 264 patients were then randomly separated into disjoint training and testing sets, with 175 and 89 patients, respectively (see Methods). The MIMIC dataset was used for primary training of the model. The second cohort of patients consisted of 115 ICU patients (after excluding patients with invalid records, see Methods) from the UCLA Health hospital system who had ECG, PPG, and ABP waveform records. These patients were randomly separated into two disjoint groups: 28 patients for secondary fine-tuning (calibration) of the MIMIC-based model, and 87 patients for testing the method. Summary cohort demographic information is shown in Table 1.

**Table 1:** Cohort characteristics of MIMIC and UCLA data.

Characteristic	MIMIC (n = 309)	UCLA (n = 150)
Male, No. (%)	178 (56.9)	80 (53.3)
Age, mean (SD), years	63.4 (16.2)	46.5 (20.1)
BMI, mean (SD), kg/m^2^	30.3 (9.3)	24.9 (4.7)
Height, mean (SD), cm	168.7 (10.4)	172.8 (11.1)
Weight, mean (SD), kg	85.0 (25.6)	73.8 (19.0)
Systolic BP, mean (SD), mmHg	106.4 (13.5)	102.6 (11.9)
Diastolic BP, mean (SD), mmHg	57.9 (11.5)	54.1 (9.1)
Mean BP, mean (SD), mmHg	74.8 (12.1)	71.3 (8.9)

**Figure 1:**
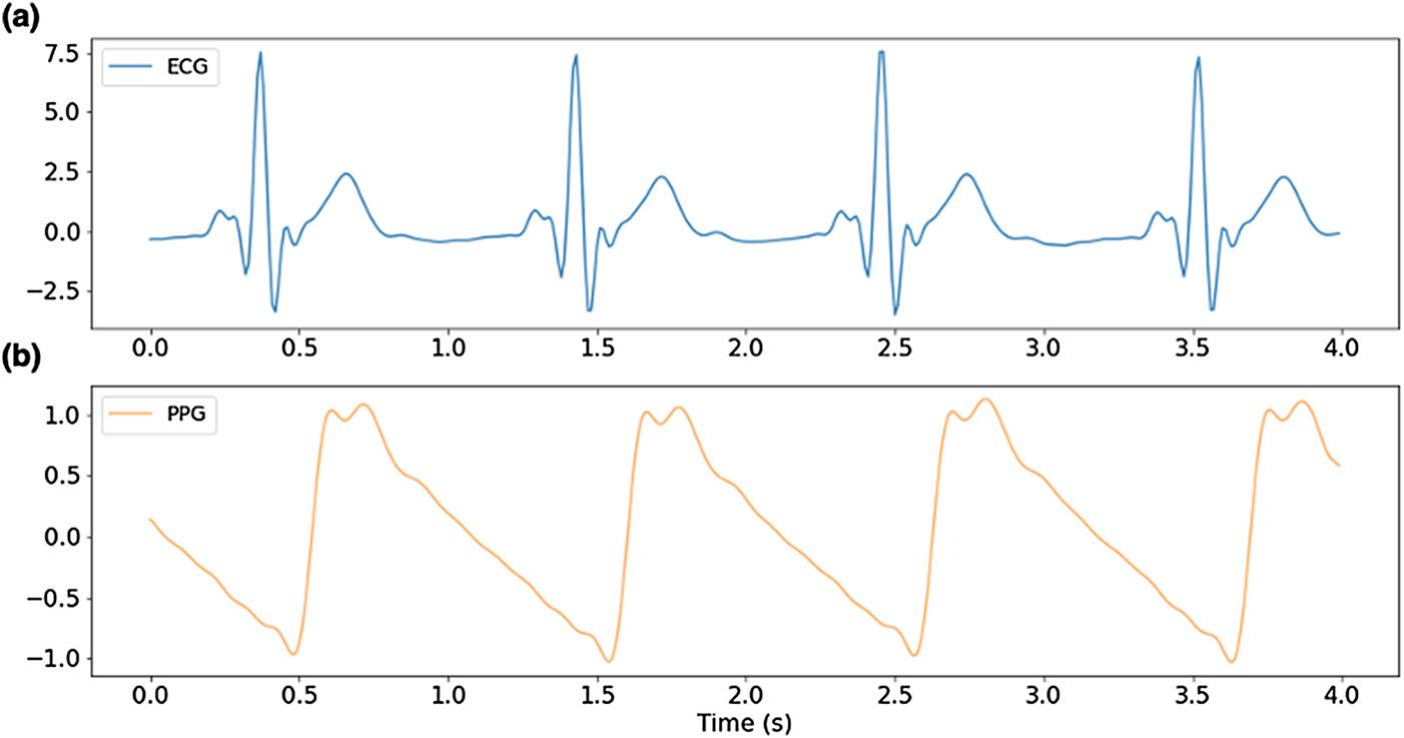
Examples of input waveforms for 1D V-Net model. (**a**) 4-s sample of electrocardiogram (ECG) waveform and (**b**) a 4-s sample photo-plethysmograph (PPG) waveform.

**Figure 2:**
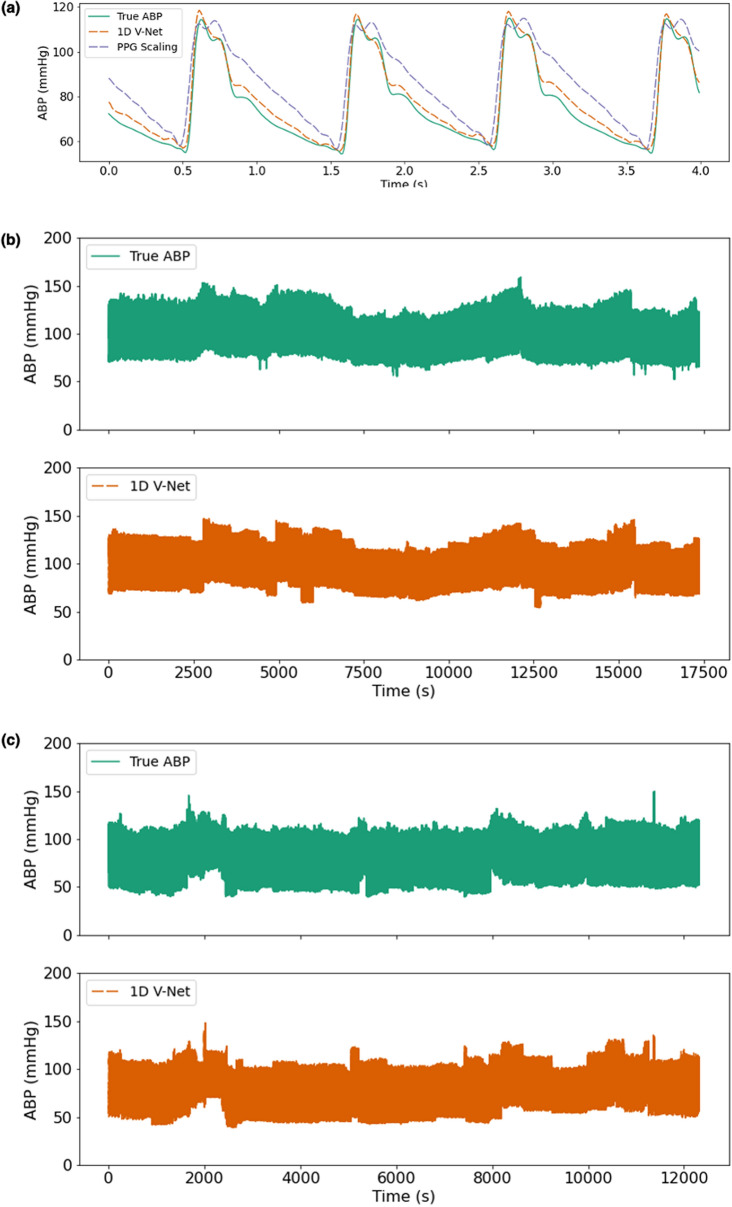
Example ground truth & predicted waveforms. (**a**) 4-s window (for the input data shown in Fig. 1) and > 3 h records (**b**,**c**). The true continuous blood pressure waveform is shown above in green, and the predicted blood pressure waveform shown below in red.

The ECG and PPG waveforms, the most recent NIBP measurements, the time since the most recent NIBP measurement, the pulse arrival time, and the heart rate were used as input to a deep learning model that was trained to predict the continuous blood pressure waveform that occurred during the timeframe of the input window. An example window of ECG and PPG waveforms used as input to the algorithm is shown in Fig. 1. The true blood pressure waveform and the predicted waveform that corresponds to the window shown in Fig. 1 are shown in Fig. 2a. Figure 2b,c show examples of how the predicted ABP waveform compares to the arterial line for much longer timeframes.

### Waveform quality evaluation

One of the unique features of our approach is that it provides a prediction of the continuous blood pressure waveform, and not simply summary statistics such as systolic or diastolic blood pressure in a window. To evaluate the quality of the waveform generated by 1D V-Net, we compared the predicted ABP waveform to the ground-truth ABP waveform obtained from the arterial line. Time was split into windows of constant duration (32 s). For measuring the method performance, we used the root mean square error (RMSE) and the correlation between the true and the predicted signals within each window. As shown in Table 2, for both cohorts, we observed a low RMSE value (MIMIC RMSE 5.823 mmHg, 7.8% of true MAP; UCLA RMSE 6.961 mmHg, 9.8% of true MAP) when comparing the true and predicted waveforms. Additionally, the correlation between the arterial line waveform and the 1D V-Net predicted waveform was high across both sets of patients (MIMIC correlation 0.957, UCLA correlation 0.947) (see Table 3).

**Table 2:** Root mean square error (mean (95% CI)) for each cohort.

Method	MIMIC	UCLA
PPG scaling	6.895 (6.876–6.914)	9.108 (9.078–9.137)
Sideris et al	13.940 (13.901–13.978)	13.111 (13.072–13.151)
1D V-Net	5.823 (5.806–5.840)	6.961 (6.937–6.985)

**Table 3:** Correlation (mean (95% CI)) between true and predicted blood pressure for each cohort.

Method	MIMIC	UCLA
PPG Scaling	0.938 (0.938–0.938)	0.926 (0.925–0.926)
Sideris et al	0.939 (0.939–0.939)	0.940 (0.940–0.940)
1D V-Net	0.957 (0.957–0.957)	0.947 (0.947–0.948)

**Figure 3:**
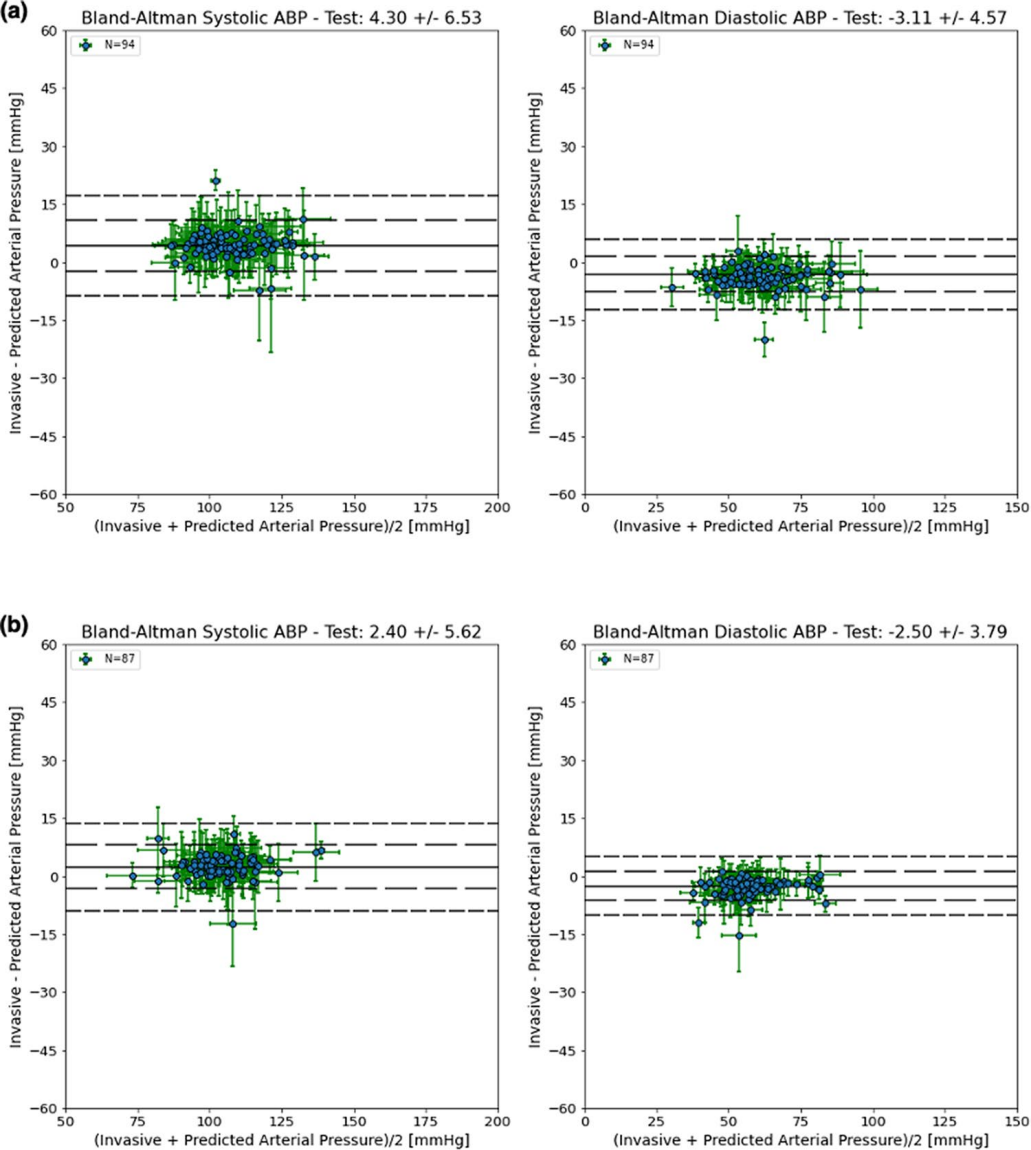
Bland–Altman plots for the MIMIC and UCLA ICU test cohorts. Systolic BP measurements per patient (left), and Diastolic BP measurements per patient (right) using a thirty-two second window; horizontal error bars represent the standard deviation of the blood pressure values, vertical error bars represent the standard deviation of the differences; solid lines indicate the mean difference values, dashed lines indicate the mean difference values +/− 1 and 2 times the standard deviation of the differences. Results for MIMIC are shown in (**a**), and UCLA in (**b**).

As an additional waveform quality analysis, we compared the systolic and diastolic values derived from the arterial line and the predicted ABP waveforms. For each heartbeat in a window, we computed the systolic and diastolic blood pressure, and compared those values to the imputed blood pressure waveform generated by our algorithm. In Fig. 3, Bland–Altman plots were used to show the differences between the predicted and true measurements across a range of blood pressure values (see Methods) for each patient in the MIMIC test set (Fig. 3a) and UCLA test set (Fig. 3b). The algorithm’s predicted waveform accurately tracks the true blood pressure values in both the MIMIC cohort (mean difference systolic BP 4.297 ± 6.527 mmHg, diastolic BP mean difference − 3.114 ± 4.570 mmHg) and the UCLA cohort (mean difference systolic BP 2.398 ± 5.623 mmHg, diastolic BP − 2.497 ± 3.785 mmHg). In both cohorts, the 1D V-Net model performance meets the Association for the Advancement of Medical Instrumentation (AAMI) criteria with mean differences less than 5 ± 8 mmHg.

### Method comparison

Next, we compared the performance of 1D V-Net with the performance of the long short-term memory (LSTM) model described in Sideris et al. and with PPG scaling. PPG scaling uses the PPG waveform as a template shape and scales the magnitude of the PPG signal in a given window to match the most recent systolic and diastolic NIBP measurements. We observe that 1D V-Net achieves the lowest RMSE and the highest correlation across both cohorts (see Tables 2 and 3) and, additionally, the PPG scaling performs better than the LSTM model for both metrics. To demonstrate the improvement in the imputed waveform quality, in Supplemental Fig. 4 we compared the residual error for PPG scaling and 1D V-Net in a 4-s window. The waveform generated by the 1D V-Net model provides not only a more accurate prediction of the systolic and diastolic values, but also the overall waveform shape compared to the PPG scaling.

Table 4 contains the distribution of differences between the true and predicted systolic and diastolic BP for each method across both cohorts (see also Supplemental Figs. 1, 2, 3 for the Bland–Altman plots that correspond to Table 4). In the MIMIC cohort, the PPG scaling method (mean difference systolic BP 6.133 ± 6.870 mmHg, diastolic BP mean difference − 4.848 ± 4.975 mmHg) performed better than the LSTM model (mean difference systolic BP 11.474 ± 13.020 mmHg, diastolic BP mean difference − 12.821 ± 11.174 mmHg), and 1D V-Net outperformed both of the other methods. Similarly, in the UCLA cohort, the PPG scaling method (mean difference systolic BP 2.668 ± 5.692 mmHg, diastolic BP mean difference − 3.595 ± 3.978 mmHg) was more accurate than the LSTM model (mean difference systolic BP 8.899 ± 11.418 mmHg, diastolic BP mean difference − 15.620 ± 9.154 mmHg), and 1D V-Net outperformed both methods. Notably, all three methods underestimate the systolic blood pressure, and overestimate the diastolic blood pressure on average. This is likely a result of the “regression to the mean” effect, which would cause predicted systolic values to trend downward to mean blood pressure, and diastolic values to trend upward to the mean blood pressure.

**Table 4:** Bland–Altman accuracy and precision (mean (95% CI) +/− SD (95% CI)) for each cohort.

	Method	MIMIC	UCLA
Systolic BP	PPG Scaling	6.133 (6.128–6.139) ± 6.870 (6.864–6.876)	2.668 (2.662–2.674) ± 5.692 (5.684–5.699)
	Sideris et al	11.474 (11.462–11.486) ± 13.020 (13.011–13.029)	8.899 (8.887–8.912) ± 11.418 (11.409–11.427)
	1D V-Net	4.297 (4.291–4.303) ± 6.527 (6.522–6.533)	2.398 (2.392–2.404) ± 5.623 (5.616–5.629)
Diastolic BP	PPG Scaling	− 4.848 (− 4.852–4.843) ± 4.975 (4.970–4.981)	− 3.595 (− 3.600–3.591) ± 3.978 (3.973–3.983)
	Sideris et al	− 12.821 (− 12.831–12.811) ± 11.174 (11.166–11.182)	− 15.620 (− 15.630–15.610) ± 9.154 (9.146–9.162)
	1D V-Net	− 3.114 (− 3.118–3.110) ± 4.570 (4.565–4.576)	− 2.497 (− 2.501–2.493) ± 3.785 (3.781–3.789)

### Dependence of model on NIBP measurements

As the model is intermittently calibrated with NIBP measurements, we wanted to measure the error as a function of time since the most recent NIBP value was obtained. Since the LSTM model does not rely on NIBP measurements at all, the RMSE remains relatively constant over time. However, the LSTM model has the highest RMSE of all methods (13.940 (95% CI 13.901–13.978) mmHg for MIMIC, 13.111 (95% CI 13.072–13.151) mmHg for UCLA). Both the PPG Scaling and 1D V-Net do see a minor increase in error as time from the NIBP measurement increases (see Supplemental Figs. 5 and 6 for MIMIC and UCLA cohorts, respectively). However, our algorithm achieves a lower RMSE compared to both the Sideris et al. model (mean difference 8.05 ± 0.45 for MIMIC, 6.13 ± 0.27 for UCLA) and PPG Scaling (mean difference 1.07 ± 0.05 for MIMIC, 2.14 ± 0.02 for UCLA) across all time points.

## Discussion

We have presented a novel method for imputing the arterial blood pressure waveform that is continuous, non-invasive, accurate, and does not require any additional hardware beyond what is standard monitoring in the acute care setting (ECG, pulse oximeter, non-invasive blood pressure cuff). This predicted waveform would allow clinicians to monitor blood pressure continuously in patients that otherwise receive blood pressure measurement intermittently. With this continuous and non-invasive monitoring, clinicians would be able to rapidly identify changes in patient state and intervene, which is crucial when even a short span of hypotension or hypertension can lead to poor health outcomes. Additionally, we have developed a preprocessing pipeline that leverages machine learning to identify windows containing low-quality signals with high precision and sensitivity. The preprocessing pipeline can be generally useful in applications that utilize physiological waveforms.

Our approach leverages the V-Net deep learning architecture (see Supplementary Note 1), which has been previously used successfully for image segmentation. Deep learning techniques have led to the development of many predictive models focusing on diagnostic applications in medicine^21–24^, resulting in promising applications including diagnosis tools for diabetic retinopathy^21^, skin cancer^22^ and arrhythmia detection using electrocardiograms (ECG)^24^. Notably, the majority of methods using deep learning in medicine have focused on classification problems, such as diagnosis, and not regression. Our approach differs from previous approaches, since it provides regression results as opposed to diagnosis, and to the best of our knowledge, the modified 1D V-Net approach has never been applied to physiological waveforms in this context.

The vast majority of ICU patients would benefit from the proposed system. Since two-thirds of patients in the ICU do not receive continuous blood pressure monitoring, imputing the ABP waveform allows clinicians to better monitor these patients without the need for any additional devices. The proposed system utilizes measurements that are currently part of the standard of care for all ICU patients, and therefore would not disrupt the current clinical workflows. Furthermore, in the remaining one-third of patients that do receive invasive blood pressure monitoring, the imputed waveform can be utilized as a secondary data source in case of instrument failure or technical artifacts, or might obviate the need for an invasive monitor that may cause complications.

An additional advantage of a continuous blood pressure monitoring system based on machine-learning software is that the model has the potential to be further improved over time with additional training data. Hospitals will continue to collect data on patients that undergo invasive blood pressure monitoring and this data can be used to update the model. This would allow the model to learn any hospital-specific instrumentation or patient population differences. For device-based continuous and non-invasive blood pressure monitoring, the performance is most often fixed at the time of deployment and cannot be updated or improved over time. Therefore, a machine-learning based software approach is at an advantage.

While other computational methods for predicting beat-to-beat blood pressure measurements have been developed, they predict only the systolic/diastolic blood pressure measurements and not the actual *continuous* waveform. Methods for imputation of systolic and diastolic blood pressure non-invasively utilize handcrafted features including pulse transit time^12, 15^, heart rate^12, 15, 16^, perfusion index^12^, stiffness index^12^, reflection index^15^, systolic/diastolic volume^15^, and PPG intensity ratio^17^. These features are then used as input to machine learning models^11–18^. The arterial blood pressure waveform can be used to estimate important cardiac parameters such as stroke volume (SV), cardiac output (CO), cardiac power output (CPO), vascular resistance, and pulse pressure variation, which can only be calculated using the ABP waveform, not the beat-to-beat measurements. Measurements like CPO are clinically relevant, and have been shown to be predictive of outcomes such as mortality^25, 26^. Additionally, the imputed blood pressure waveform can be used as input to predictive algorithms which use the arterial blood pressure waveform as input, such as predicting hypotensive events up to 15 min before they occur^27^. Another limitation of previous studies is the limited number of patients used to train and evaluate models. The small sample sizes, most often only several dozen patients and from a single health system, calls into question the generalizability of the methods. To demonstrate the wide applicability of our proposed method, we used data from over four hundred patients and two separate health systems to show that our model successfully predicts the continuous blood pressure waveform in new patients.

## Methods

This manuscript follows the “Guidelines for Developing and Reporting Machine Learning Predictive Models in Biomedical Research: A Multidisciplinary View”^28^.

### Study participants and sampling procedures

The first retrospective, de-identified dataset consisted of 309 randomly sampled ICU patients with ECG (lead II) waveforms, photo-plethysmographic (PPG) waveforms, arterial blood pressure (ABP) waveforms, and at least one non-invasive blood pressure measurement from the MIMIC-III waveform database^20, 29^ matched subset. The 309 patients were randomly divided into a training set (206 patients, 66% of the cohort) and a testing set (103 patients, 33% of cohort). Of these patients, 31 patients from the training set and 14 from the testing set were removed because none of the data were found to pass the quality filtering process (see Dataset Creation), leaving 175 patients in the training set and 89 in the testing set. The second retrospective dataset consisted of 150 randomly sampled UCLA ICU patients with recorded ECG, PPG, and ABP waveforms. The UCLA dataset was divided into 40 patients for calibrating the model, and 110 patients for testing the model. Of these patients, 12 were removed from the calibration set and 23 were removed from the testing set due to issues with waveform data quality, for a final training set of 28 patients and testing set of 87 patients.

The ABP waveform prediction model was first trained using patients from the MIMIC training set, and model performance results were computed using patients from the held-out MIMIC testing set. Then, the model parameters were fine-tuned using patients from the UCLA calibration set, and model performance results were computed using patients from the held-out UCLA testing set.

### Dataset creation

#### Demographic data

Cohort demographic data were extracted for the MIMIC patients using the MIMIC clinical database. The features extracted included patient age, height, weight, BMI and sex. For de-identification, patients older than 90 have their age encoded as 300 in the clinical database. Since we do not know the exact age of these patients, we set their age to 90 years old. The same demographic information for the UCLA patients was retrieved from the UCLA Clinical Data Mart, a data warehouse system that extracts data from UCLA's electronic medical record system (EPIC Systems, Madison, WI, USA). As was done for the MIMIC data, any UCLA patients older than 90 years had their age set to a maximum of 90 years for de-identification purposes.

#### Normalization of waveforms

In both cohorts, if the signal sampling rate was greater than 100 Hz, each waveform signal was downsampled to 100 Hz. Each signal was low-pass filtered with a cutoff frequency of 16 Hz to remove high-frequency noise. Since the range of the ECG and PPG signals differed for each patient, we scaled each 32-s window by subtracting the running median and dividing by the difference between the upper and lower quartiles.

#### Derived non-invasive blood pressure

Intermittent non-invasive blood pressure (NIBP) measurements were extracted for each patient from the MIMIC-III database. However, since NIBP measurements were only recorded, on average, once per hour, the frequency of non-invasive blood pressure measurement was insufficient. Therefore, we created derived NIBP measurements by sampling the invasive blood pressure waveform (i.e. median systolic, diastolic BP, and mean ABP in a 4-s window) every 5 min to simulate the frequency of NIBP measurement that would be used when deploying the algorithm in practice. Since the derived non-invasive blood pressure was measured every 5 min, but the waveforms were sampled at 100 Hz, we used the most recent derived NIBP measurement to fill in missing NIBP values. As an additional feature, we also included the time (in milliseconds) from the most recent NIBP measurement to each sample in our input window.

#### Correction of signal drift

As mentioned by the authors of the MIMIC dataset^30^, issues with clock synchronization can cause waveform signals to drift. We corrected for signal drift between the PPG signal and the arterial blood pressure signal by computing the cross-correlation of the PPG signal under consideration with the arterial blood pressure signal. Once the cross-correlation was computed, the location of the highest cross-correlation was used to correct the PPG signal drift by shifting the signal in time, up to a maximum of 4 s in either direction.

#### Creation of valid windows

After completing the above preprocessing steps, we selected valid 32-s windows from the record using a sliding window approach with a 16-s step size. A window size of 32 s was chosen since it is long enough to give temporal context for several heartbeats, yet short enough to ensure that there would be a sufficient number of windows that did not contain artifacts. See Table 5 for filtering rate across each cohort. For each 32-s window, the following process was used to determine whether the window will be included in the development or validation of the algorithm.

**Table 5:** Window filtering statistics for each cohort.

	MIMIC	UCLA
Total minutes, No., mins	1,535,413	240,241
Valid minutes, No., mins	115,388	35,601
Total heartbeats, No	9,791,870	2,935,846
Total windows per patient, median (IQR)	8376. (4509.3–16,656.3)	4087.0 (2627.5–5414.5)
Valid windows per patient, median (IQR)	411.5 (90.8–1076.0)	254.0 (67–743)
Total record length per patient, median (IQR), mins	4467.2 (2404.9–8883.3)	2179.7 (1401.3–2887.7)
Valid record length per patient, median (IQR), mins	219.5 (48.4–573.9)	135 (35.7–396.5)
Median (IQR), %	4.8 (1.1–12.9)	8.5 (2.2–24.9)
Mean (SD), %	9.6 (12.4)	15.5 (17.3)
Min/Max %	0.01/63.8	0.02/71.5

#### Filtering of windows with artifacts

Each waveform (ECG, PPG, ABP) was checked for signal quality and windows with technical artifacts or invalid parameters were removed. Windows were excluded if they met any of the following criteria for the ECG or PPG data: the variance of the signal was less than a small value (1e−4 for ECG and PPG), the number of peaks in a window was greater than a threshold (4 peaks/sec × 32 s for both ECG, PPG; 4 peaks/sec results in maximum allowable HR of 240 beats/min), or the number of peaks in a window was less than a threshold (0.5 peak/sec × 32 s for ECG, PPG; 0.5 peak/sec results in minimum allowable HR of 30 beats/min).

For the arterial blood pressure waveform, windows were excluded if they met any of the following criteria: the mean signal value was less than 30 mmHg or greater than 200 mmHg, the maximum signal value was greater than 300 mmHg or less than 60 mmHg, the minimum signal value was less than 20 mmHg, the variance of the signal was less than 80, we could not find any systolic or diastolic blood pressure values using the “find_peaks” function from the scipy^31^ Python package, the difference between two consecutive systolic or diastolic values was greater than 50 mmHg, the waveform signal was flat (i.e. did not change value for 2 or more consecutive samples), a pulse pressure value in the window was greater than 70 mmHg, the difference between the systolic BP and the most recent NIBP was greater than 40 mmHg, or the time delay between a diastolic blood pressure measurement and the subsequent systolic blood pressure measurement was greater than 0.5 s.

If a window contained signals that passed all of the above criteria, we performed two additional filtering steps, and excluded windows that failed either of these criteria: the number of PPG peaks was different than the number of arterial blood pressure peaks, or the mean absolute time difference between arterial blood pressure peaks and PPG peaks was greater than 0.15 s (after correcting for signal drift). Finally, outlier windows were excluded if the mean ECG, PPG, or ABP signal in the window was greater than the 99.9% quantile or less than the 0.01% quantile.

Assuming a window under consideration passed the above criteria, it was included in our training or testing dataset. The input features were then scaled to have a mean of zero and standard deviation of one using a running mean and standard deviation.

#### Filtering with PPG quality index

Since the quality of the PPG waveform is crucial to the performance of our algorithm, we developed an additional filtering step to remove windows containing artifacts in the PPG signal. A CNN model was trained to classify four-second PPG windows as “valid” or “invalid” using the PPG waveform as input. To train the model, 4000 four-second PPG windows from the 206 patients in the MIMIC training set were hand-labeled (by B.L.H.) as “valid” (2682, 67.1%) if the window was free of artifacts, or “invalid” (1318, 32.9%) if the window contained artifacts, using visual inspection. From these 4000 windows, we randomly sampled a subset of 100 windows and an expert clinician (M.C.) then labeled the windows to estimate the initial classification quality. In 94% of the sampled windows, the clinician’s classification matched the initial labeling (Cohen’s kappa: 0.857). We then trained a 3 layer CNN model (see Supplemental Note 2 for additional details) on 70% of the patients to predict the window classification. The model’s predicted probability of being a valid window was then used as a quality index (QI) to exclude windows highly likely to contain artifacts. The remaining 30% of patients (separate from the training patients) from the MIMIC training set were held out for model validation. The quality index threshold for filtering was chosen as the minimum threshold (value: 0.811) achieving a positive predictive value (precision) of at least 0.95 in the validation set. This method of setting the threshold was chosen to reduce the number of windows containing artifacts (false positives) when training and testing the ABP waveform prediction models, while minimizing the threshold to be as sensitive as possible. Since the ABP waveform prediction model uses thirty-two second windows as input, yet the PPG QI model uses four-second windows, the PPG QI model was applied to the eight non-overlapping four-second windows contained within a thirty-two second window, generating a total of eight PPG QI values per window. The minimum PPG QI value in a thirty-two second ABP prediction window was used to determine if the signal quality was greater than or less than the PPG QI filtering threshold.

#### Waveform features

To improve the ABP waveform imputation we included two features derived from the non-invasive signals that have been previously shown to be predictive of blood pressure values: pulse arrival time (PAT) and heart rate (HR)^12, 15, 16^. Calculation of the pulse arrival time was achieved by first identifying the ECG R wave using the peak finding algorithm implemented in the scipy^31^ Python package, and then identifying the PPG systolic peaks using the peak finding algorithm. The number of seconds between the ECG R wave peak and the subsequent PPG systolic peak were then calculated. PAT values were excluded from a window if the value was deemed to be unreasonably small (< 0.1 s) or unreasonably large (> 1.0 s). Finally, for each window, the median (log-transformed) and standard deviation of the PAT were used as two additional input feature channels. If PAT values were excluded or unavailable, the missing values were imputed using the median observed value.

Additionally, the HR was calculated for each window using the PPG signal. The scipy peak finding algorithm was similarly used to detect the PPG systolic peaks. Then, the number of these peaks found in a given window was divided by the window length (in seconds) and multiplied by the number of seconds in a minute to give the resulting HR in beats per minute. The HR was also included as an additional input feature channel for each window. Any missing HR values were imputed using the median observed value.

#### Comparison with other methods

The performance of two other methods were used as a comparison: PPG scaling and the LSTM model of Sideris et al.^18^.

#### PPG scaling

Previous work has shown the utility of the PPG signal for predicting blood pressure measurements^11–18^. These approaches use manual feature extraction^11, 12, 14, 17^ or learn features from the data using machine learning methods^13, 15, 16, 18^. Since the PPG waveform is correlated with the ABP waveform, one approach for predicting the ABP waveform uses the PPG waveform as a template shape and scales the magnitude of the PPG signal in a given window to match the most recent systolic and diastolic pseudo-NIBP measurements. Specifically, the PPG waveform is stretched such that the maximum value in the window is equal to the most recent systolic NIBP measurement, and the minimum value in the window is equal to the most recent diastolic NIBP measurement. The PPG window was scaled using the transformation

$$PPG_{scaled } = \left( {PPG - min\left( {PPG} \right)} \right)\frac{{NIBP_{sys} - NIBP_{dias} }}{{max\left( {PPG} \right) - min\left( {PPG} \right)}}_{{}} + NIBP_{dias}$$

#### LSTM model

Sideris et al.^18^ proposed training a patient-specific LSTM model (i.e. one model per patient) to impute the ABP waveform, using the PPG signal from the same window of time as input. While the results were promising, a critical issue is that the model requires that each patient first receive invasive blood pressure monitoring so a patient-specific model can be trained. However, this means that only a fraction of the patient population can benefit from such a model (the subpopulation that receives invasive blood pressure monitoring), and only after they have already undergone invasive monitoring. Therefore, the algorithm may not generalize to the majority of the patient population who do not receive invasive monitoring. To fairly compare the LSTM model to our proposed model, we trained the model as described in the paper, using 128 nodes as a default since the number of nodes was not described.

#### Algorithm development

We developed a deep learning model that takes as input a window of two signals, ECG and PPG, and several constant values encoded as additional channels by repeating the value for each timestep: the most recent non-invasive systolic, diastolic, and mean blood pressure measurements prior to the window, the time since the most recent NIBP measurement, the median and standard deviation of the pulse arrival time, and heart rate. The model is trained to minimize the residual difference between the PPG scaling method and the true ABP waveform to compensate for the difference in waveform morphology, as well as the change in BP over time. This forces the network to focus on windows where the PPG scaling significantly differs from the ABP waveform, and therefore improve on the PPG scaling method. With enough data and a large enough model, the neural network should be able to similarly learn the scaling method. However, to accelerate the learning process we designed the method to learn the residual error. The model output is a prediction of the residual difference between the continuous ABP waveform and the baseline PPG scaling waveform, and this predicted residual difference is added to the PPG scaling waveform to generate the 1D V-Net waveform prediction. The deep learning model architecture was based on the V-Net CNN architecture, which has been proven to be useful in the field of image segmentation^19^ (see Supplemental Note 1 for description). However, instead of 2D or 3D image segmentation, we leveraged the V-Net architecture for 1D signal-to-signal transformation. The motivation behind the V-Net architecture is that it learns a compressed representation of the input data to identify global features, and then reconstructs the signal from this representation. During the reconstruction process, local features are learned to modify the waveform at a finer scale. Our architecture is the same as described in the V-Net paper^19^, except instead of 3D volumes with multiple channels our data is represented as a 1D signal with multiple channels. Otherwise, the architecture (number of layers, convolutions per layer, kernel size, etc.) remained the same. An additional L2 penalty was added to the activation of the final network layer to force the network to prioritize modification of the PPG scaling residual waveform.

To train the network, we used a custom loss function consisting of two parts. The first was the mean squared error between the true ABP waveform and the predicted waveform, which forces the network to learn an accurate prediction of the entire waveform. The second part of the loss was the mean squared error between the true and predicted waveforms at the locations of the systolic and diastolic points, to encourage the network to be particularly accurate at these locations.

The deep-learning model was implemented using Keras^32^ and was trained for a maximum of 100 epochs using random weight initialization and the Nadam^33^ optimizer with default parameters beta1 of 0.9, beta2 of 0.999. The learning rate used was 0.001 with a schedule decay of 0.004, and the mini-batch size was 32. For the last layer of the network, an L2 activation penalty with weight 0.0005 was added. Ten percent of the training data was held out for validation, and these data were used for choosing hyperparameters. If the validation loss did not improve after 8 epochs, the model training process was stopped early.

### Algorithm evaluation

#### Bland–Altman

To evaluate the agreement between the gold standard invasive blood pressure measurements (the arterial catheter) and the DNN predictions, we used the Bland and Altman method^34^ as this is the standard method for comparing the agreement of two medical devices. Accuracy and precision of the predictions were described as the mean ± standard deviation of the differences between the predicted and true blood pressure values, and the differences are considered acceptable by the Association for the Advancement of Medical Instrumentation (AAMI) criteria if less than 5 ± 8 mmHg. The method was implemented as follows. For each window under consideration, we extracted the systolic and diastolic blood pressure measurements from the ABP waveform using the peak finding algorithm described previously. These measurements were used as the gold standard reference values. We then used the peak finding algorithm on the DNN-generated waveform to determine the systolic and diastolic blood pressure measurements and used these as the comparison values. If the number of systolic or diastolic points identified by the peak finding algorithm differed between the true and predicted waveforms, we performed local alignment by minimizing the sum of the differences between the indices of the true and predicted waveform points. We then took the difference between the reference blood pressure measurement and the predicted blood pressure measurement pairs, and plotted these differences as a function of the average of the reference and predicted value pairs. The 95% confidence intervals (CI) were calculated using bootstrapping.

#### Waveform metrics

Two metrics were used to quantitatively compare the quality of the predicted waveform and the true ABP waveform: root mean square error (RMSE) and correlation. These values were calculated for all windows per patient. The 95% confidence intervals (CI) were calculated using bootstrapping.

#### Error as a function of time since NIBP measurement

RMSE was calculated as a function of time from the most recent NIBP measurement. The time between the window and the most recent NIBP measurements were binned into ten second intervals, and for each bin the mean and SD of the RMSE between the true and predicted waveforms was calculated.

#### Ethical approval and patient consent

The institutional review board (IRB) of the Massachusetts Institute of Technology (Cambridge, MA) and Beth Israel Deaconess Medical Center (Boston, MA) approved the use of MIMIC-III for research, and the requirement for individual patient consent was waived because the project did not impact clinical care and all protected health information was de-identified^20^. Retrospective data collection and analysis was approved by the UCLA IRB. All research was conducted in accordance with the tenets set forth in the Declaration of Helsinki.

## Supplementary Information

Supplementary Information.

## Data Availability

Access to the MIMIC-III database can be requested at https://mimic.physionet.org/. The UCLA datasets generated during and/or analyzed during the current study are not publicly available due to institutional restrictions on data sharing and privacy concerns.
