# Supplementary material for: Imputation of the continuous arterial line blood pressure waveform from non-invasive measurements using deep learning
Source: Sci Rep. 2021 Aug 3;11:15755. doi: 10.1038/s41598-021-94913-y (PMC8333060; doi:10.1038/s41598-021-94913-y)

# Supplemental Material

### Supplemental Note 1: V-Net Architecture

The V-Net architecture, introduced by Milletari et al.[^1^](https://www.zotero.org/google-docs/?zwRvrX)^9^, has been proven to be an effective method for segmentation of 2D and 3D images. The architecture includes a compression stage, where the image resolution is consecutively reduced using a number of convolutional layers (downsampling), and a decompression stage, where the image resolution is recovered using the same number of de-convolutional layers (upsampling) (see Supplemental Figure 10). This compression stage allows the network to learn global features in the compressed representation. With the decompression stage, the network learns to localize the features that were identified in the compressed representation. The compression stage reduces the resolution by using a kernel stride size of two, which effectively downsamples the signal by a factor of two (similar to a traditional pooling operation used in CNNs). While the signal is downsampled by a factor of two, the number of features (channels) extracted increases by a factor of two.

Residual connections are used between the convolutional and de-convolutional layers at the same depth of each stage. These residual connections force the network to learn a residual function, which accelerates the convergence process. Use of residual connections is further motivated by the similarity of the PPG waveform to the ABP waveform, as demonstrated by the performance of the PPG scaling method. Since the shape of the two waveforms is relatively similar, our model learns to predict the difference rather than learn a more complex transformation of the PPG waveform that matches the ABP waveform. However, rather than solely rely on the PPG waveform for predicting the ABP waveform, our model additionally incorporates features extracted from the ECG waveform. This serves two primary purposes: it provides additional information to supplement the PPG waveform, and it allows the method to be more robust to signal artifacts that may occur in one or both waveforms, by leveraging a combination of the two.

### Supplemental Note 2: PPG QI Model

The CNN model consisted of 3 convolutional blocks, followed by a fully-connected layer (see Supplemental Figure 7 for architecture diagram). Each convolutional block consisted of a convolutional block with 64 filters, followed by batch normalization, an ReLU activation, and max pooling. The first convolutional block had a filter width of 15, and the remaining convolutional blocks had filter widths of 9. The model was trained with a batch size of 32, using the Nadam optimizer with a learning rate of 0.0002. If the validation ROC AUC did not improve after 5 epochs, the model training process was stopped early.

Of the 4000 labeled PPG windows, 2682 (67.1%) were labeled as valid and 1318 (32.9%) were labeled as invalid. From these 4000 windows, we randomly sampled a subset of 100 windows and an expert clinician (M.C.) then labeled the windows to estimate the initial classification quality. In 94% of the sampled windows, the clinician’s classification matched the initial labeling (Cohen’s kappa: 0.857). These 100 windows were also relabeled by B.H. to estimate the intra-rater agreement. The relabeling agreed with the initial labeling in 97% of the cases (Cohen’s kappa: 0.930). The median number of windows per patient was 7 (IQR: 4-13). On the held-out validation patients, the PPQ QI model achieved an ROC AUC of 0.920 (0.905-0.934), PR AUC of 0.954 (0.938-0.968), Precision of 0.951 (0.937-0.964), and Recall of 0.768 (0.744-0.793). See Supplemental Figure 8 for ROC and Precision-Recall curve plots.

### Supplemental Figure 1. Bland-Altman Plots (per patient) - V-Net

Systolic BP measurements per patient (left), and Diastolic BP measurements per patient (right) using a thirty-two-second window size; horizontal error bars represent the standard deviation of the blood pressure values, vertical error bars represent the standard deviation of the differences; solid lines indicate the mean difference values, dashed lines indicate the mean difference values +/- 1 and 2 times the standard deviation of the differences. (a) MIMIC (b) UCLA


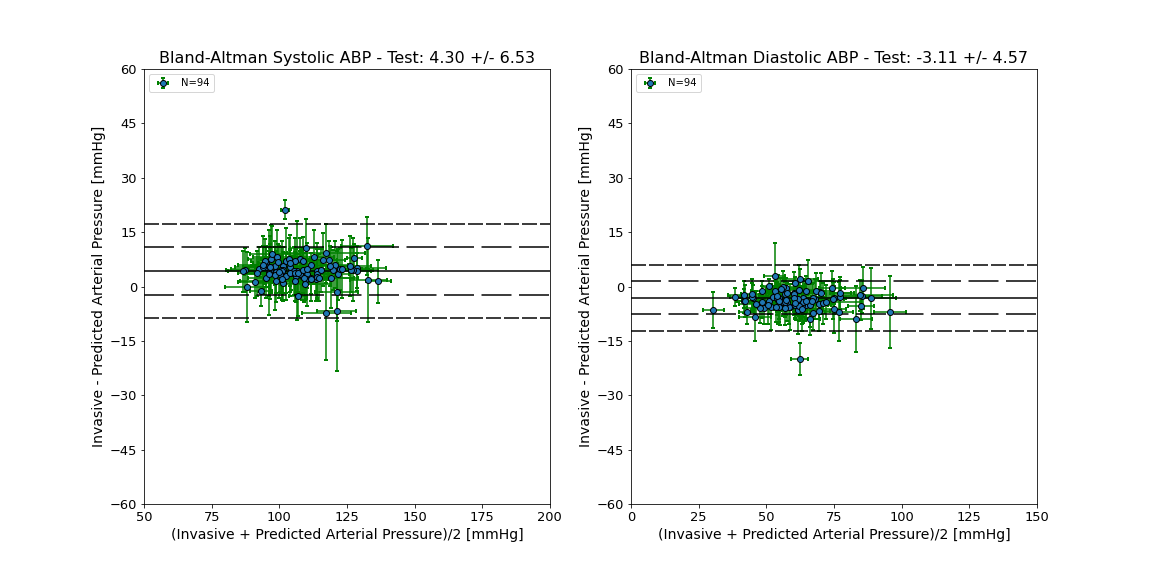


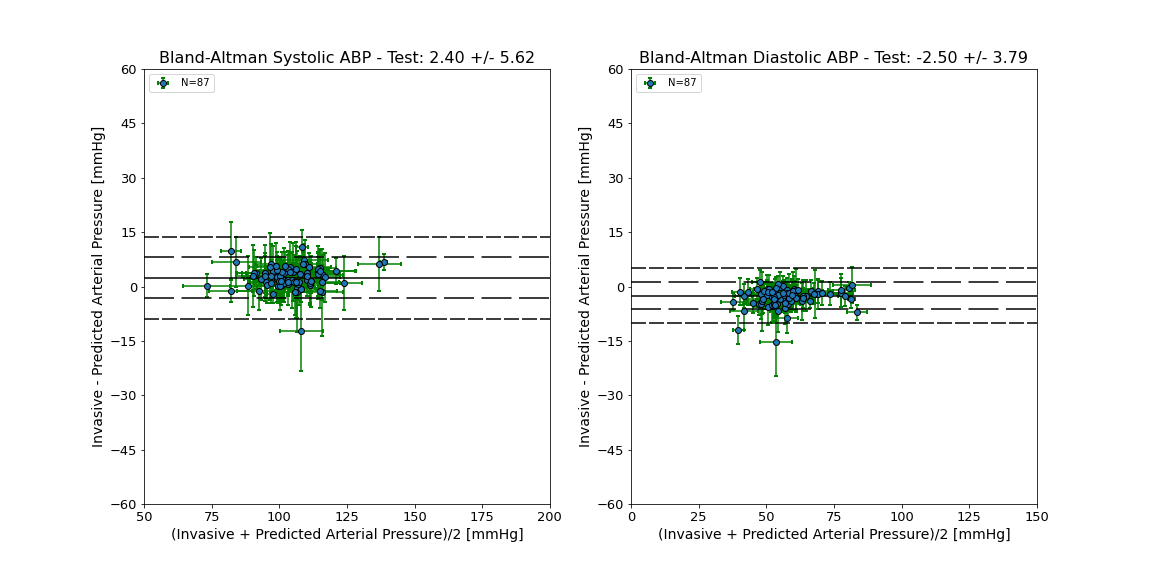


### Supplemental Figure 2. Bland-Altman Plots (per patient) - PPG Scaling

Systolic BP measurements per patient (left), and Diastolic BP measurements per patient (right) using a thirty-two second window size; horizontal error bars represent the standard deviation of the blood pressure values, vertical error bars represent the standard deviation of the differences; solid lines indicate the mean difference values, dashed lines indicate the mean difference values +/- 1 and 2 times the standard deviation of the differences. (a) MIMIC (b) UCLA


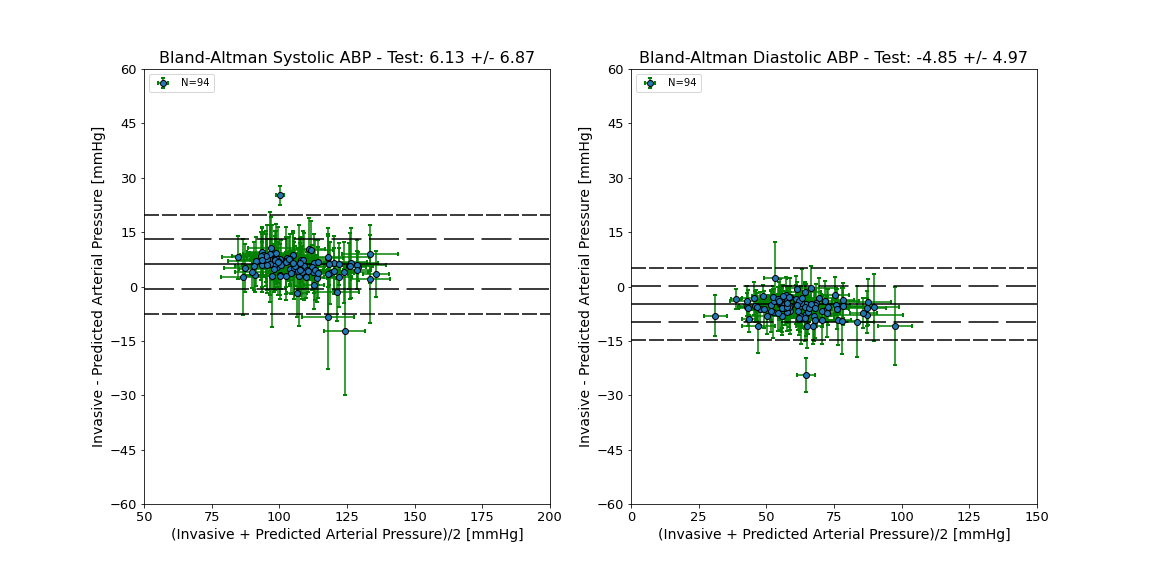


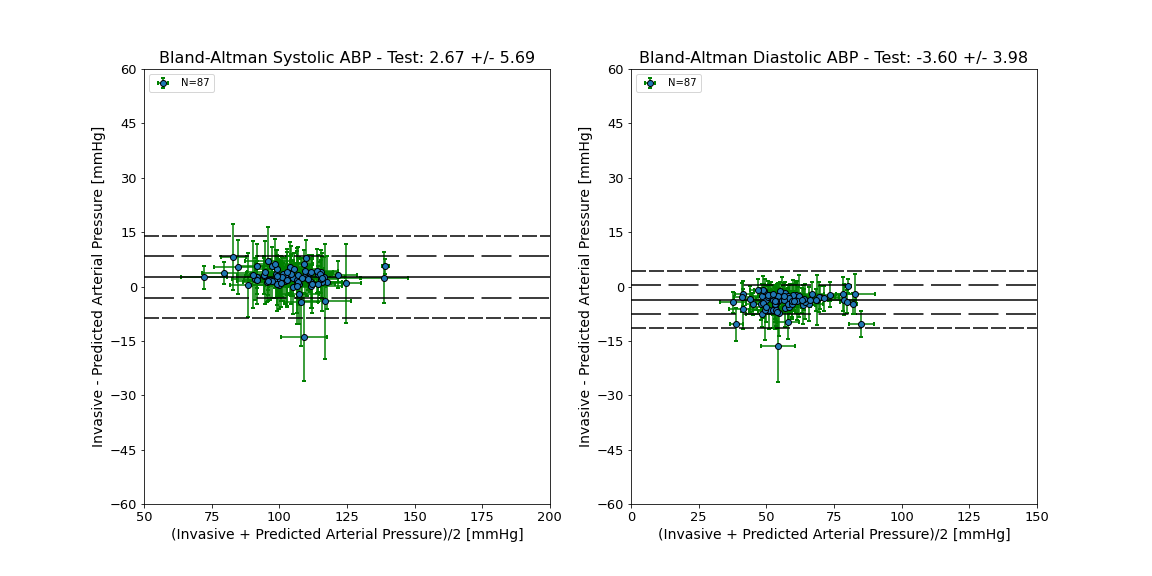


### Supplemental Figure 3. Bland-Altman Plots (per patient) - Sideris et al.

Systolic BP measurements per patient (left), and Diastolic BP measurements per patient (right) using a thirty-two second window size; horizontal error bars represent the standard deviation of the blood pressure values, vertical error bars represent the standard deviation of the differences; solid lines indicate the mean difference values, dashed lines indicate the mean difference values +/- 1 and 2 times the standard deviation of the differences. (a) MIMIC (b) UCLA


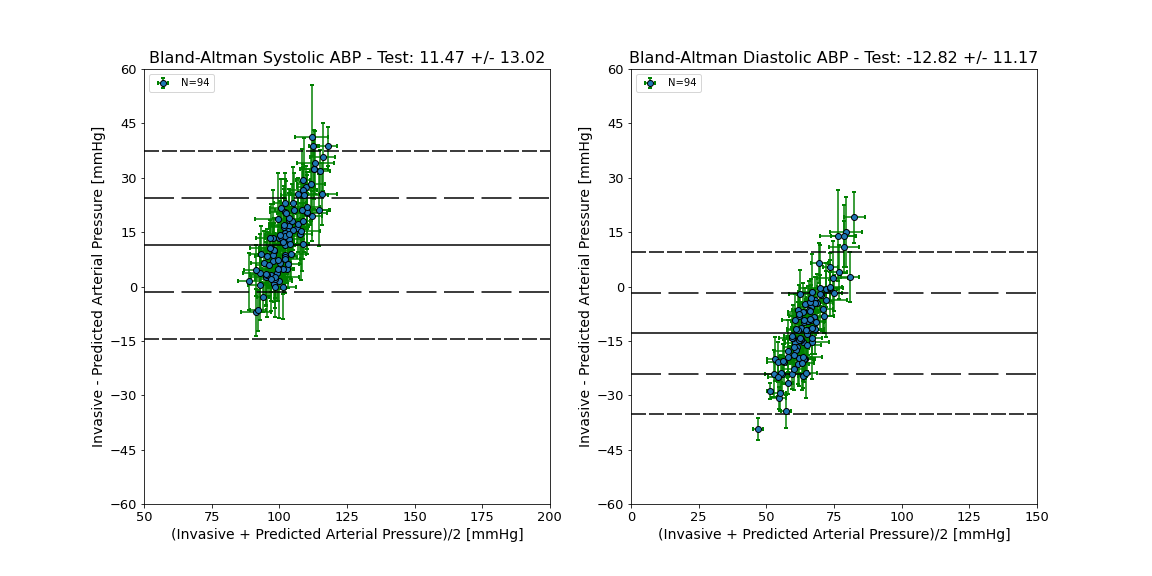


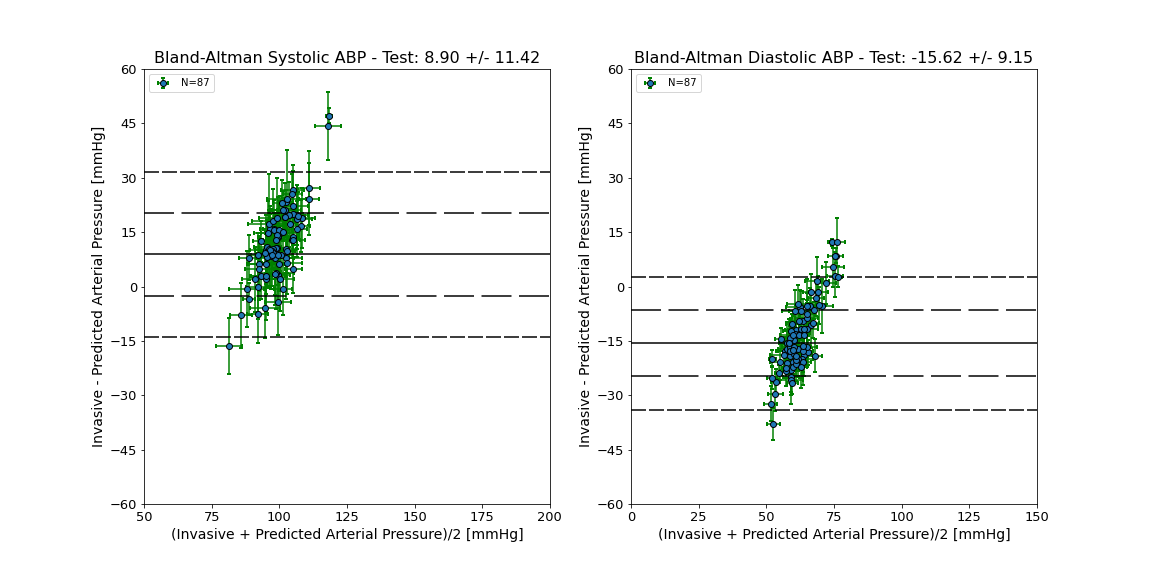


### Supplemental Figure 4: Residual error over time

Example of the residual error (i.e. the difference) for a 4-second window. The residual error between the continuous ABP waveform and the scaled PPG waveform is shown by the dashed green line. The residual error between the continuous ABP waveform and the 1D V-Net predicted waveform is shown by the dashed red line. The black dotted line represents no residual error (i.e. a perfect ABP waveform prediction). Systolic and diastolic points are annotated with crosses and circles for the PPG scaling and 1D V-Net predictions, respectively.

###
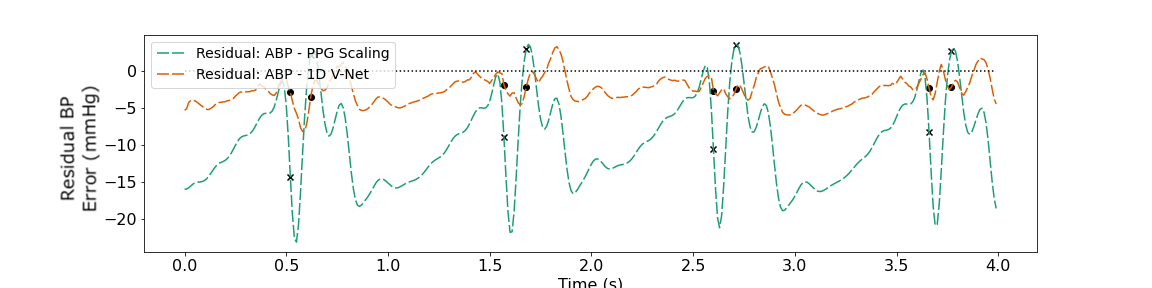


### Supplemental Figure 5. RMSE as a function of time from most recent NIBP measurement - MIMIC

Root mean square error (RMSE) was calculated as a function of time from the most recent NIBP measurement using the MIMIC testing set. The time between the window and the most recent NIBP measurements were binned into ten second intervals, and for each bin the mean and SD of the RMSE between the true and predicted waveforms was calculated.


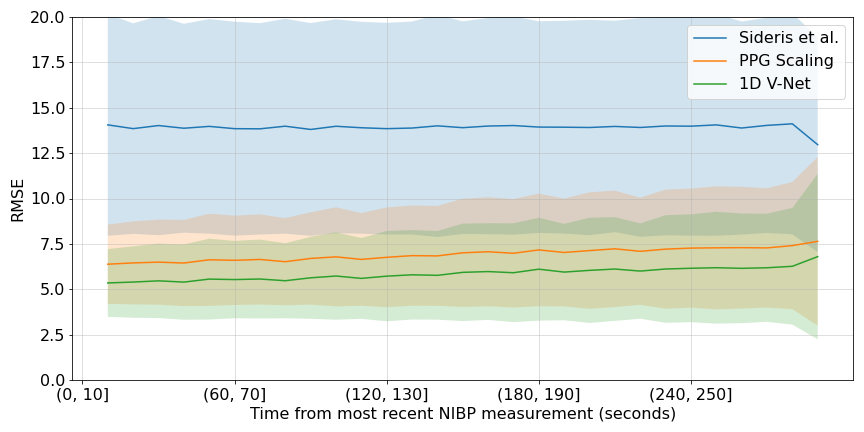


###

###

### Supplemental Figure 6. RMSE as a function of time from most recent NIBP measurement - UCLA

Root mean square error (RMSE) was calculated as a function of time from the most recent NIBP measurement using the UCLA testing set. The time between the window and the most recent NIBP measurements were binned into ten second intervals, and for each bin the mean and SD of the RMSE between the true and predicted waveforms was calculated.


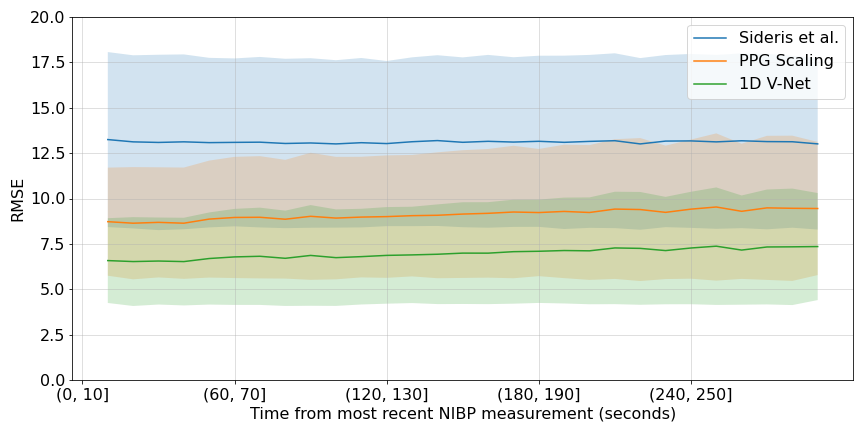


### Supplemental Figure 7: PPG QI CNN Model Architecture

The CNN model architecture is depicted, broken down by individual layers. The corresponding layer input and output shapes are shown along with the layer type, where the first shape value corresponds to the batch size, the second number generally refers to the number of time steps (samples), and the third number is the number of filters.

###
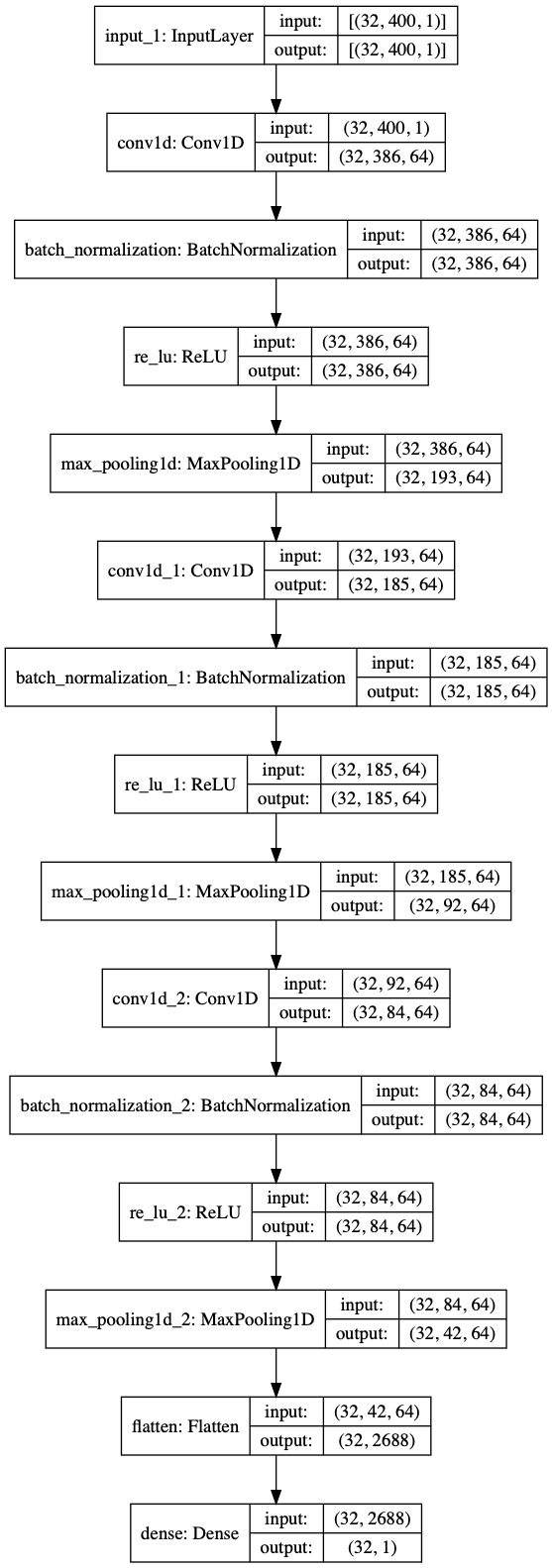


###

### Supplemental Figure 8: ROC and Precision-Recall Curves for PPG QI Model

ROC curves (left) show the false positive rate on the x-axis and the true positive rate on the y-axis. The optimal point is the upper-left corner. Precision-recall curves (right) show the recall (sensitivity) on the x-axis and precision (positive predictive value, or PPV) on the y-axis. The optimal point is in the upper-right corner. Shown below are the results of the PPG QI model on the held-out validation set patients.

###
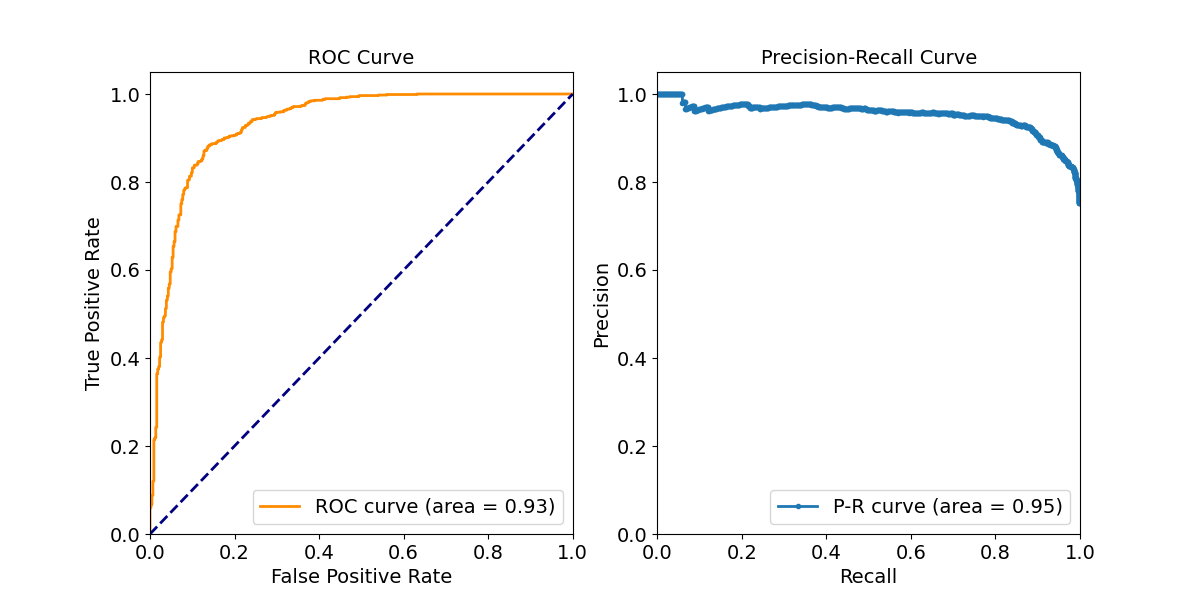


###

### Supplemental Figure 9: Preprocessing and Training Pipeline Diagram

Flow diagram depicts how the MIMIC and UCLA datasets were split into training, testing, and calibration sets. The MIMIC dataset was divided into two separate sets (training and testing) such that patients only appeared in the train set or the test set, not both. The MIMIC train set was used to fit the models, and the MIMIC test was then used to evaluate the model performance. Similar to the MIMIC dataset, the UCLA dataset was also divided into two separate sets of patients (calibration and testing) such that patients only appeared in either the calibration set or the test set, not both. Both the MIMIC dataset and the UCLA dataset were processed using the same preprocessing pipeline and artifact filtering process. The models that were fit using the MIMIC training set were then further trained (calibrated) on the UCLA calibration set. Finally the UCLA test set was used to evaluate the calibrated models.


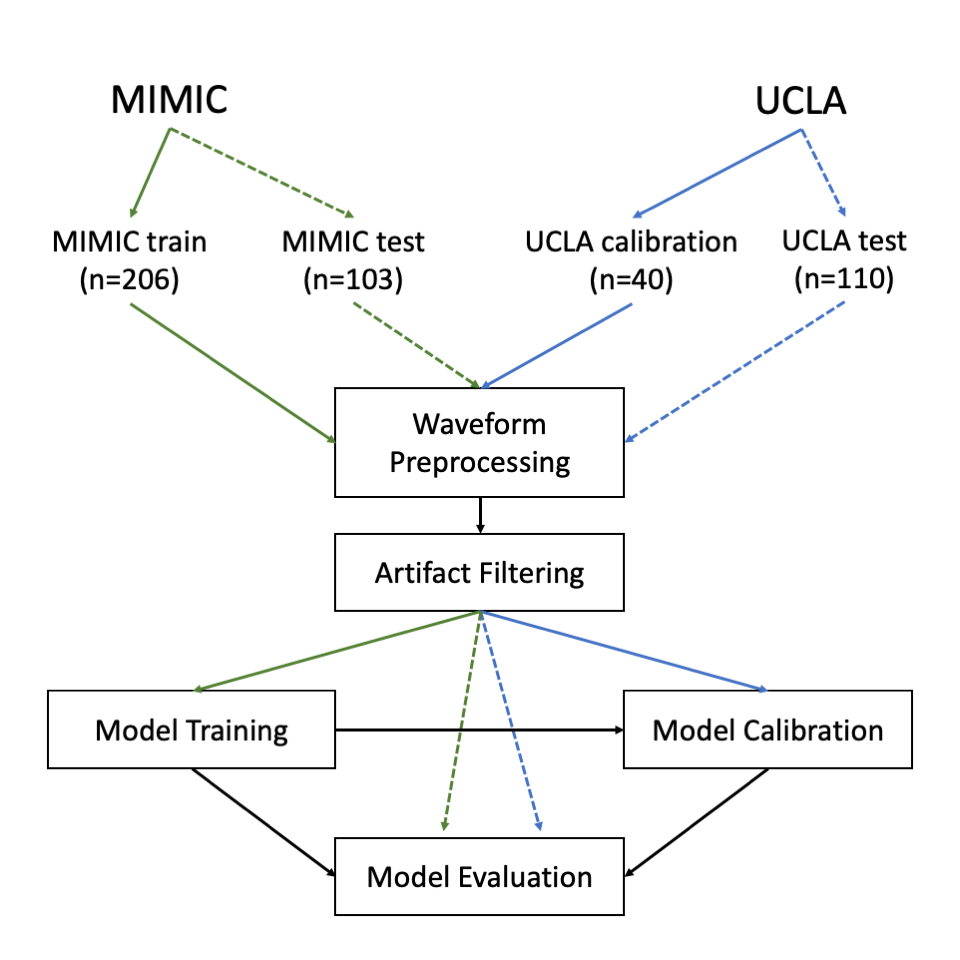


###

### Supplemental Figure 10: 1D V-Net Model Architecture Diagram

Diagram depicting the V-Net model architecture, introduced by Milletari et al.[1](https://www.zotero.org/google-docs/?zwRvrX)9 and adapted for 1D waveform signals. Arrows depict the forwarding of the output of each block/layer. For a detailed breakdown of residual convolutional block architecture, see Milletari et al.[1](https://www.zotero.org/google-docs/?zwRvrX)9


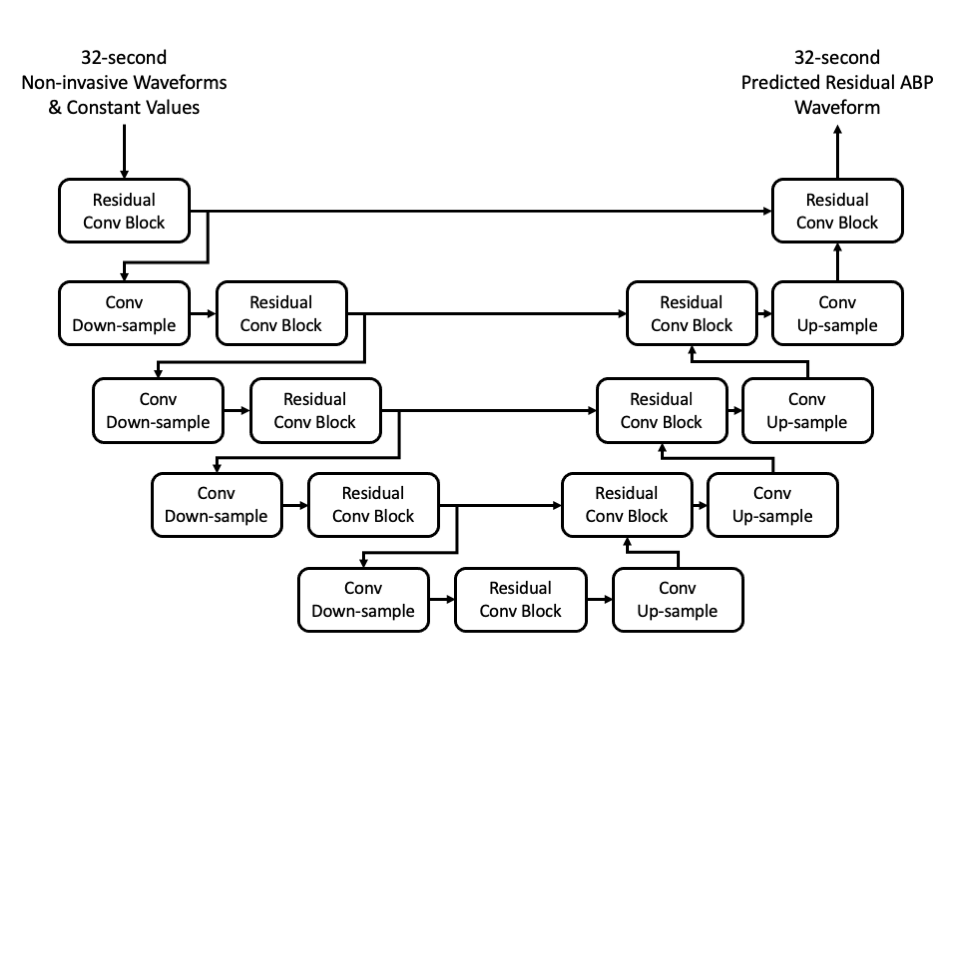

Supplement: Supplementary file 1 — Supplementary Information. [file 41598_2021_94913_MOESM1_ESM.docx]
